# Supplementary material for: Effects of Porphyra tenera Supplementation on the Immune System: A Randomized, Double-Blind, and Placebo-Controlled Clinical Trial
Source: Nutrients. 2020 Jun 2;12(6):1642. doi: 10.3390/nu12061642 (PMC7352330; doi:10.3390/nu12061642)
Supplement: Supplementary file 1 [file nutrients-12-01642-s001.pdf]

Supplemental files list

Table S1. URI incidence in study subjects during the 8-week intervention period.

Symptoms		PTE group (n=8)				Placebo group (n=2)				
		**Baseline**	**Wk 4**	**Wk 8**	***p*-value[1]**	**Base line**	**Wk 4**	**Wk 8**	***p*-value[1]**	***p*-value[1]**
**Total Score**		3.0±2.2	6.0±1.4	1.5±0.7	0.125	3.0±0.0	2.00	4.00	-	0.030[2]
**Sore throat**	score			0.5±0.7	0.247					0.417
	duration			0.5±0.7	0.247					0.417
**Rhinorrhea**	score	-	1.0	0.00	-	0.5±0.7	1.00	1.00	0.939	0.132
	duration	-	4.5±3.5	0.00	0.045	2.5±3.5	15.00	4.00	0.111	0.037[3]
**Nasal congestion**	score	-	-	-	-	0.5±0.7	0.00	0.00	0.808	0.324
	duration	-	-	-	-	2.5±3.5	0.00	0.00	0.245	0.040[4]
**Sneezing**	score	0.3±0.5	1.5±0.7	0.00	0.055	1.0±0.0	1.00	2.00	-	0.022[5]
	duration	0.5±1.0	4.0±1.4	0.00	0.017	4.0±1.4	10.00	2.00	0.140	0.056[6]
**Hoarseness**	score	0.5±0.6	0.00	0.00	0.363	-	-	-	-	0.569
	duration	4.5±7.1	0.00	0.00	0.556	-	-	-	-	0.365
**Myalgia**	score	0.3±0.5	0.00	0.00	0.680	-	-	1.00		0.086
	duration	1.0±2.0	0.00	0.00	0.680	-	-	2.00		0.199
**Fever**	score	0.3±0.5	0.00	0.00	0.680	-	-	-		0.823
	duration	1.3±2.5	0.00	0.00	0.680	-	-	-	-	0.701
**Headache**	score		1.5±2.1	0.00	0.247					0.328
	duration		1.5±2.1	0.00	0.247					0.328
**Cough**	score	1.0±0.8	1.5±0.7	0.5±0.7	0.487	0.5±0.7	0.00	0.00	0.939	0.576
	duration	8.0±1.2	2.5±0.7	1.0±1.4	0.630	1.5±2.1	0.00	0.00	0.975	0.583
**Sputum**	score	0.8±0.5	0.5±0.7	0.5±0.7	0.842	0.5±0.7	0.00	0.00	0.934	0.984
	duration	4.3±4.2	1.0±1.4	1.0±1.4	0.453	1.5±2.1	0.00	0.00	0.975	0.465

Values are presented as means ± SDs

[1] Analyzed by linear mixed model

[2] Multiple comparison by Bonferroni correction; Test 2nd -3rd vs Placebo 2nd -3rd: *p*=0.0194

[3] Multiple comparison by Bonferroni correction; Test 1st-2nd vs Placebo 1st-2nd : *p*=0.0330, Test 2nd -3rd vs Placebo 2nd-3st: *p*=0.0354

[4] Multiple comparison by Bonferroni correction; Test 1st-2nd vs Placebo 1st-2nd : *p*=0.0485, Test 1st-3rd vs Placebo 1st -3rd : *p*=0.0485

[5] Multiple comparison by Bonferroni correction; Test 2nd -3rd vs Placebo 2nd -3rd : *p*=0.0152

[6] Multiple comparison by Bonferroni correction; Test 2nd -3rd vs Placebo 2nd -3rd : *p*=0.0370

Table S2. Laboratory profiles of the subjects in this study

Laboratory profiles (standard range)	PTE group(n=58)			Placebo group (n=53)			
	Baseline	Week 8	p-value [1]	Baseline	Week 8	p-value [1]	p value[2]
WBC (4.8–10.8×10^3/μL)	5.4±1.2	5.0±1.1	0.0004	5.6±1.2	5.3±1.1	0.0041	0.3978
RBC (4.2–5.4×100^3/μL)	4.4±0.4	4.3±0.4	0.0002	4.4±0.4	4.3±0.4	0.0203	0.3298
Hemoglobin (12–16g/dL)	13.5±1.3	13.3±1.3	0.0042	13.5±1.1	13.3±1.0	0.0353	0.5025
Hematocrit (37–47%)	40.0±3.7	39.3±3.6	0.0019	39.9±3.1	39.4±2.7	0.0338	0.5193
Platelet (130–450×10^3/μL)	244.3±57.8	234.3±48.4	0.0015	244.2±42.3	241.6±44.8	0.5034	0.1259
Neutrophil (50-75%)	52.7±8.6	54.2±8.8	0.0966	51.6±8.2	53.2±7.3	0.0819	0.9796
Lymphocyte (20~44%)	37.9±7.5	35.9±8.0	0.0213	38.5±8.0	36.9±6.8	0.0416	0.7374
Monocyte (2~9%)	6.7±1.5	7.1±1.7	0.0563	7.0±1.5	7.0±1.3	0.6175	0.0838
Eosinophil (~5%)	2.1±1.4	2.1±1.3	0.5201	2.2±1.7	2.2±1.8	0.8893	0.8547
Basophil (~2%)	0.7±0.3	0.7±0.3	0.1267	0.7±0.3	0.7±0.4	0.0575	0.6226
ESR (Male~9mm/hr Female:~20mm/hr)	18.8±12.0	21.815.5	0.0272	18.8±11.6	18.9±12.5	0.9538	0.1215
hs-CRP (~5mg/L)	0.5±1.0	1.6±6.3	0.1553	1.2±2.8	0.7±1.3	0.2359	0.0698
GGT (8–48IU/L)	18.9±11.1	20.9±17.3	0.0954	20.9±15.3	20.1±10.8	0.5245	0.1084
AST (12–33IU/L)	23.2±5.6	24.7±5.2	0.0188	23.3±4.4	24.1±5.1	0.2992	0.4855
ALT (5–35IU/L)	21.9±8.2	23.3±7.3	0.0699	22.1±6.9	23.1±5.7	0.2469	0.7284
Total bilirubin (0.2–1.2mg/dL)	0.8±0.3	0.8±0.3	0.5996	0.9±0.3	0.9±0.3	0.3303	0.2867
Total protein (6.7–8.3g/dL)	7.0±0.3	7.0±0.4	0.7731	7.0±0.3	6.9±0.3	0.5858	0.8417
Albumin (3.5–5.3g/dL)	4.5±0.2	4.5±0.2	0.0030	4.5±0.2	4.5±0.2	0.0662	0.3215
BUN (8–23mg/dL)	14.9±3.2	15.2±3.8	0.5847	16.2±3.9	15.6±3.8	0.3761	0.3035
Creatinine (0.7–1.7mg/dL)	0.7±0.2	0.7±0.2	0.2050	0.7±0.2	0.7±0.2	0.0010	0.1574
Glucose (74–106mg/dL)	87.6±9.5	86.6±11.5	0.3468	91.5±9.3	88.7±9.2	0.0031	0.1642
CK (50~200 IU/L)	100.7±44.2	105.0±51.0	0.4514	111.7±59.7	126.4±110.4	0.3110	0.5027

LDH (218~472 IU/L)	405.0±53.9	410.9±47.0	0.3237	396.7±49.2	412.3±55.3	0.0070	0.2334
pH(urine) (4.5~9.0)	6.4±1.1	6.3±1.0	0.5570	6.3±1.0	6.3±1.1	0.9015	0.6018

Values are presented as mean ± SD

1 Analyzed by paired *t* test

2 Analyzed by Linear Mixed Model between groups

Abbreviations: WBC,White Blood Cell; RBC, Red Blood Cell; ALP; Alkaline Phosphatase; GGT, Gamma Glutamyl Transferase; AST;Aspartate Transaminase; ALT,Alanine Transaminase; BUN, Blood Urea Nitrogen; CK, creatine kinase; LDH, lactate dehydrogenase.
